# Preablation Stimulated Thyroglobulin/TSH Ratio as a Predictor of Successful I^131^Remnant Ablation in Patients with Differentiated Thyroid Cancer following Total Thyroidectomy

**DOI:** 10.1155/2014/610273

**Published:** 2014-04-09

**Authors:** Syed Zubair Hussain, Maseeh uz Zaman, Sarwar Malik, Nanik Ram, Ali Asghar, Unaib Rabbani, Nida Aftab, Najmul Islam

**Affiliations:** ^1^Section of Endocrinology, Department of Medicine, Aga Khan University Hospital, Stadium Road, P.O. Box 3500, Karachi 74800, Pakistan; ^2^Section of Nuclear Medicine, Department of Radiology, Aga Khan University Hospital, P.O. Box 3500, Karachi 74800, Pakistan; ^3^Department of Community Health Sciences, Aga Khan University Hospital, Stadium Road, P.O. Box 3500, Karachi 74800, Pakistan; ^4^Dow Medical College, P.O. Box 942, Karachi 74200, Pakistan

## Abstract

*Background*. About 90% of thyroid cancers are differentiated thyroid cancers. Standard treatment is total thyroidectomy followed by radioactive I^131^remnant ablation and TSH suppression with thyroxine. Unsuccessful ablation drastically affects the prognosis of patients with DTC particularly high risk individuals; therefore, identifying the factors that affect the success of ablation is important in the management of patients with DTC. sTg is a good predictor of successful ablation in DTC. Its levels can be influenced by tumor staging and TSH values, as well as other factors. Therefore, we did this study using TSH to correct the predictive value of sTg in success of RRA. * Methods*. We retrospectively reviewed the records of 75 patients with DTC, who underwent total thyroidectomy followed by RRA and TSH suppression. * Results*. Preablation sTg and sTg/TSH ratio are significantly associated with ablation outcome. Cutoff value for sTg to predict successful and unsuccessful ablation was 18 ng/mL with 76.7% sensitivity and 79.1% specificity, while for sTg/TSH cutoff was 0.35 with 81.4% sensitivity and 81.5% specificity (*P* < 0.001). Association was stronger for sTg/TSH ratio with adjusted odds ratio (AOR) 11.64 (2.43–55.61) than for sTg with AOR 5.42 (1.18–24.88). * Conclusions*. Preablation sTg/TSH ratio can be considered as better predictor of ablation outcome than sTg, tumor size, and capsular invasion.

## 1. Introduction

Thyroid cancer is the most common endocrine tumor. About 90% of thyroid cancers are differentiated (papillary or follicular) thyroid cancers [1]. Overall the prognosis in patients with differentiated thyroid cancers (DTC) is good [2]. With appropriate treatment, the majority of these patients get cured except in patients with metastasis where the prognosis is worse [2, 3]. Management of patients with differentiated thyroid cancer involves total thyroidectomy followed by radioactive I^131^remnant ablation (RRA), except in patients with intrathyroidal tumor ≤ 1 cm in size [4, 5]. The RRA may also be avoided in low risk well-differentiated thyroid cancer with postsurgical stimulated thyroglobulin (sTg) < 1 ng/mL, an approach not yet approved by American Thyroid Association (ATA) [6–8]. The theoretical goals of adjuvant ablation are (a) to destroy any residual microscopic disease, (b) to enhance sensitivity of diagnostic whole body iodine scan (WBIS) and specificity of serum thyroglobulin (Tg) which facilitates follow-up and early detection of recurrence or metastatic disease, and (c) to use postablative WBIS which is more sensitive than diagnostic WBIS for detection of nodal or distant functioning metastases [9]. According to ATA guidelines, sTg and neck ultrasound with or without diagnostic WBIS should be done 6–12 months after RRA in these patients to see the presence or absence of disease [4].

An area of ongoing controversy is the optimal dose of RAI required to successfully ablate remnant tissue after total or near-total thyroidectomy with a single administration in low risk group. This can be achieved by either dosimetry method introduced by Benua et al. or by fixed empiric activity of RAI [10]. According to Maxon et al., about 30,000 rad (300 Gy) radiation dose to thyroid bed was required for successful ablation and Bal CS and Padhy found 50 mCi (1850 MBq) of RAI to deliver this required dose to thyroid bed after total thyroidectomy [11, 12]. However, due to cumbersomeness of dosimetry method, the majority of treating physicians have adopted fixed empiric activity of RAI (30–200 mCi or 1110–7400 MBq) with no consensus about adequate ablative dose in low risk patients [12]. Due to these variable results, there has been lack of consensus among various thyroid organizations on the optimum RAI dose for remnant ablation in low risk group. The British Thyroid Association's 2007 guidelines recommend the use of high dose RAI [13]. National Comprehensive Cancer Network (NCCN; 2010 guidelines), the American Thyroid Association (ATA; 2009 guidelines), and European Thyroid Cancer Task Force consensus report (ETCTF; consensus report, 2006) advise that clinicians can choose between the low dose and the high dose due to lack of reliable evidence from large randomized studies [14].

Tg is a well-recognized marker in the follow-up of patients with differentiated thyroid cancers after thyroidectomy for persistent disease, distant metastasis, or disease recurrence [15–17]. sTg before RRA is a good predictor of successful ablation in these patients [15, 18]. Its levels can be influenced by tumor staging and TSH levels as well as other factors. Therefore we did this study using TSH to correct the predictive value of sTg in success of RRA. Our objective was to see whether sTg/TSH ratio can be used as a good predictor of successful RRA and to compare its predictability with sTg.

## 2. Patients, Material, and Methods

*Patients*. We retrospectively reviewed the medical record of all patients with DTC treated at the Section of Endocrinology, Aga Khan University Hospital, Karachi, from 2003 to 2013. An approval was obtained from Ethical Review Committee of Aga Khan University Hospital for this study. Only 75 patients met our inclusion criteria.

Our inclusion criteria were as followed: (1) all adult patients with DTC; (2) those who underwent total thyroidectomy; (3) those having stimulated TSH (sTSH), sTg, and Anti-Tg antibodies (Anti-Tg) 3-4 weeks after thyroidectomy without thyroxine replacement; (4) those who received RRA; (5)those having posttherapeutic WBIS; (6) those having neck ultrasound, diagnostic WBIS, and sTg; and (7) Anti-Tg at 6–12 months after RRA. We excluded patients with (1) less then total thyroidectomy, that is, subtotal thyroidectomy or lobectomy; (2) diagnostic WBIS done before RRA because of possible stunning effect and low sensitivity; (3) Anti-Tg > 40 IU/mL prior to RRA as it might influence the absolute value of sTg.

All 75 patients were treated as per following protocol: (1) they underwent total thyroidectomy; (2) they were without thyroxine replacement for 3-4 weeks following surgery; (3) they were on low iodine diet for 2 weeks prior to RRA; (4) they got their sTSH, sTg, and Anti-Tg done approximately 2-3 days prior to RRA; (5) they received RRA of 30 to 100 mCi in ATA low risk, 150 mCi in ATA intermediate risk, and 200 mCi in ATA high risk groups; (6) they got their posttherapeutic WBIS done 3 to 5 days after RRA; (7) they were started on thyroxine after RRA to suppress TSH below 0.1 in intermediate and high risk group and below 0.5 in low risk group; and (8) they get their sTSH, sTg, Anti-Tg, neck ultrasound, and diagnostic WBIS 6–12 months after RRA. Diagnostic WBIS was performed 48 hours after giving 2 mCi of I^131^. We used dual head digital gamma camera (ECAM, Siemens, Germany) for both posttherapeutic and diagnostic WBIS.

We assessed the following variables as a possible predictor of successful ablation: sTg before RRA, sTg/TSH ratio before RRA, age, sex, RRA dose, postablative WBIS findings, TNM staging, and histology (follicular or papillary).

Successful ablation is defined as sTg < 2 ng/mL with negative Anti-Tg antibodies, no evidence of tumor on diagnostic WBIS as well as on neck ultrasound 6–12 months after RRA.

TSH was measured using Chemiluminescent assays with analytical sensitivity of 0.008 uIU/mL, functional sensitivity of 0.008 uIU/mL, and reportable range of 0.008 to 150 uIU/mL. Tg was measured using Chemiluminescent assays with analytical sensitivity of 0.2 ng/mL and functional sensitivity of 0.9 ng/mL interassay for values higher than 2 ng/mL. Anti-Tg antibodies were measured using Chemiluminescent assays with analytical sensitivity of 2.2 IU/mL, interassay precision of 4.6–5.8%, and intra-assay precision of 3.2–4.9% with reportable range of 20–3000 IU/mL and normal range < 40 IU/mL.

## 3. Statistical Analysis

Data was entered and analyzed using SPSS version 19.0. Comparative analysis was done using student's* t*-test or Wilcoxon Rank sum test for continuous variables and chi-square test for categorical variables. Cutoff values of serum preablation sTg and sTg/TSH were determined and receiver operating characteristic (ROC) curves were generated using ROC analysis. Values with maximum sensitivity and specificity were selected to define cutoff values. Univariate and multivariate logistic regression were performed to see the predictors of successful ablation. For the analysis *P* value less than 0.05 was considered significant.

## 4. Results

Records of 75 patients who fulfilled our criteria were retrieved. Out of these 60% (45) had successful ablation, while 40% (30) had unsuccessful ablation. There was no significant difference between two groups, that is, patients with successful ablation and unsuccessful ablation with respect to age and gender. Most of the tumors were papillary carcinomas 91.9% and 90% in successful and unsuccessful groups, respectively. Similarly no statistically significant differences were found between the two groups in size of tumor, capsular invasion, pathological and TNM tumor stages, lymph node involvement, ATA risk, and the dose of radioactive iodine given. However more distant metastasis was seen in unsuccessful group as compared to successful group and this difference was statistically significant. There was also significant difference in lower median levels of sTg and sTg/TSH ratio in patients with successful and unsuccessful ablation; that is, lower median level of sTg is 3.05 (0.20–14.5) among patients with successful ablation, whereas it is 140 (17.65–689) among patients with unsuccessful ablation. Similarly lower median level of sTg/TSH ratio is 0.067 (0.005–0.26) among patients with successful ablation compared to 2.84 (0.366–22.61) among unsuccessful ablation group. 76.6% of unsuccessful group patients had sTg levels > 18 ng/mL as compared to 24.4% in successful group. Similarly more of unsuccessful group patients, that is, 80% of unsuccessful group patients, had sTg/TSH ratio > 0.35 against 20.5% in successful group (Table 1).

**Figure 1:**
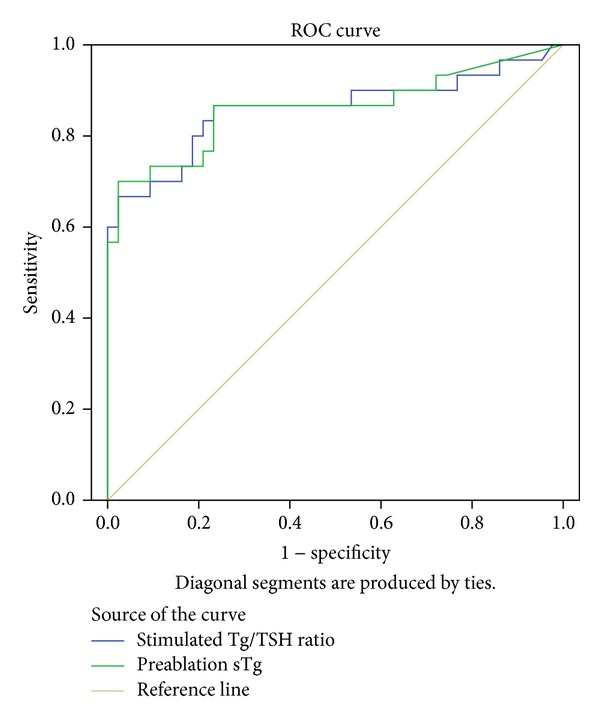
ROC curve of the accuracy of serum preablation sTg and sTg/TSH ratio in predicting unsuccessful ablation.

Cutoff value for sTg to predict successful and unsuccessful ablation was 18 ng/mL with 76.7% sensitivity and 79.1% specificity, while for sTg/TSH cutoff was 0.35 with 81.4% sensitivity and 81.5% specificity. These cutoff values were used in all the analysis (Table 2). ROC curve for accuracy is illustrated in Figure 1, showing sensitivity and specificity for different cutoff values of sTg and sTg/TSH ratio (Figure 1).

Univariate logistic regression found no significant association of ablation outcome with age, gender, tumor size, capsular invasion, distant metastasis, and ETA risk. However lymph node involvement, sTg levels > 18 ng/mL, and sTg/TSH ratio > 0.35 were significantly associated with unsuccessful ablation (Table 3). Similar trend persisted in multivariate analysis and only sTg levels and sTg/TSH ratio remained significantly associated with unsuccessful ablation. Association was stronger for sTg/TSH ratio with AOR 11.64 (2.43–55.61) than for sTg with AOR 5.42 (1.18–24.88) (Table 4).

## 5. Discussion

Unsuccessful ablation may adversely affect the prognosis of patients with DTC; therefore, identifying the factors that affect the success of ablation is very important in the management of patients with DTC.

Thyroglobulin is a large glycoprotein with molecular weight of 660,000 Daltons containing >3000 amino acids [19]. It is produced in the endoplasmic reticulum of thyroid follicular cells and stored in the Golgi apparatus to be secreted in thyroid follicular lumen [19]. TSH stimulation leads to intracellular reuptake of thyroglobulin and its proteolytic degradation into active forms of thyroid hormones, that is, triiodothyronine and thyroxine [20]. Initially Tg was thought to exist in the thyroid gland only but in 1961 its presence was demonstrated in the peripheral circulation too as a byproduct of thyroid hormone synthesis [21]. In healthy individuals, small amount of Tg is released physiologically into the circulation but in DTC it is actively released into circulation [20]. As Tg is actively released into circulation by DTC, therefore its level in circulation following total thyroidectomy is an established method of detecting recurrent or persistent disease particularly when performed after thyroxine withdrawal or recombinant TSH administration [22–26]. Measurement of serum Tg becomes an important parameter for residual or recurrent disease in the follow-up of patients with DTC following total thyroidectomy and RRA [4, 23, 24].

Predicting the outcome of ablation in patients with DTC is important as this can help make appropriate decisions for patient's management. Previous studies have focused on sTg only as a laboratory parameter for predicting the ablation outcome [16, 27]. A recent retrospective study conducted on 133 patients revealed that a preablation sTg < 8.55 ug/L predicted disease remission after 18–24 months of RRA with sensitivity of 88%, specificity of 72%, positive predictive value (PPV) of 47%, and negative predictive value (NPV) of 95% [18]. Another study conducted on 96 patients in Brazil found that patients with sTg levels < 18 ng/mL before RRA had 5.89 times greater chance of successful ablation compared to those with sTg > 18 ng/mL (*P* < 0.0001) with sensitivity of 71.4%, specificity of 70.2%, PPV of 71.4%, and NPV of 70.2% [16]. Another recent study conducted in China showed that in patients with DTC who underwent total thyroidectomy, sTg and its ratio with TSH before RRA might be considered as predictive markers for metastasis [1]. We used diagnostic WBIS as an exclusion criterion due to its low impact on the decision to ablate and because of concerns over ^131^I induced stunning of normal thyroid remnants and distant metastases from thyroid cancer [4].

This is first attempt to assess the association of sTg/TSH ratio with ablation outcome, which was found to be significantly associated with ablation outcome than sTg. In this study, factors such as age, gender, type of tumor, size of tumor, and capsular invasion were not found to be significantly associated with RRA outcome, while sTg levels are significantly associated, a finding which is consistent with other studies [1, 27–31]. Important finding of our study is association of sTg/TSH ratio with ablation outcome. We found that sTg/TSH ratio had stronger association with ablation outcome than sTg. As sTg/TSH ratio was found to be significantly associated with ablation outcome; therefore, it can help us in predicting the success of RRA and we hypothesized that it might also help in adjusting the dose of I^131^. This adjustment in the dose of I^131^remnant ablative dose might be very important particularly in low risk DTC where there is still no consensus on optimal dose.

We also defined the appropriate cutoff values for these two predictors. Cutoff value of 18 ng/mL for sTg from our study is consistent with other studies from Brazil, but our sensitivity and specificity are higher than those reported earlier for cutoff value of 18 ng/mL [16]. Since no cutoff value for sTg/TSH ratio was available; therefore, we also determined cutoff value for sTg/TSH ratio, that is, 0.35 for predicting ablation outcome. These cutoff values for sTg and sTg/TSH ratio in our study have high sensitivity and specificity in predicting the outcome.

This study is one of its kinds, investigating the association of sTg/TSH ratio with ablation outcome in patients with DTC. We found that patients with sTg/TSH ratio < 0.35 before RRA had 11.64 times greater chance of successful ablation compared to those with sTg/TSH ratio > 0.35 with sensitivity of 80.0% and specificity of 81.4% (*P* < 0.001). However limitation of our study was its retrospective nature; therefore, we could retrieve data of only 75 patients who fulfilled our inclusion criteria. Many of the previous studies regarding role of sTg in predicting RRA outcome were also retrospective in nature [8, 15, 16, 18, 29, 31]. Another limitation of this study is use of different ablative doses in low, intermediate, and high risk groups which could have an impact on success of ablation as British Thyroid Association 2007 guidelines favor use of high dose for RRA [13]. Our data do not show a significant impact of dose of RRA upon outcome and this is in concordance with two recently published prospective trials claiming no difference in outcomes in patients treated with 30 and 100 mCi RRA (32,33). Because of its smaller sample size, the result of this study should be interpreted cautiously and we recommend further studies not only to validate the cutoff levels that we have set but also to confirm the other findings of this study.

## 6. Conclusion

Our study adds to the value of preablation sTg and establishes the role of preablation sTg/TSH ratio in predicting the ablation outcome. One of the suggestions that came out from this study is that preablation sTg/TSH ratio might be used for risk stratification, as till now there is no laboratory parameter in the risk stratification of the ATA and ETA for DTC before ablation. So patients with sTg/TSH ratio >0.35 can be placed in ATA and ETA high risk categories as sTg/TSH ratio has even more significant association with RRA outcome than size and capsular invasion. However considering the limited sample size in this study, further studies are warranted to confirm these findings.

## Figures and Tables

**Table 1:** Comparison of baseline characteristics of successful and unsuccessful ablation.

Characteristic	Successful ablation % (*n*)	Unsuccessful ablation % (*n*)	*P* value
Age			
40 or less	44.4 (20)	53.3 (16)	0.45
More than 40	55.6 (25)	46.7 (14)	
Gender			
Male	42.2 (19)	43.3 (13)	0.924
Female	57.8 (26)	56.7 (17)	
Histopathological types			
Papillary carcinoma	91.9 (41)	90.0 (27)	0.871
Follicular carcinoma	8.9 (4)	10.0 (3)	
Size of tumor			
>4 cm	77.8 (35)	63.3 (19)	0.172
<4 cm	22.2 (10)	36.7 (11)	
Capsular invasion			
Yes	15.6 (7)	33.3 (10)	0.072
No	84.4 (38)	66.7 (20)	
Pathological tumor stage			
pT1	40 (18)	33.3 (10)	0.297
pT2	33.3 (15)	20.0 (6)
pT3	17.8 (8)	26.7 (8)
pT4	8.9 (4)	20.0 (6)
LN involvement			
N1a	8.9 (4)	23.3 (7)	0.064
N1b	33.3 (15)	23.3 (7)
Nx	28.9 (13)	43.3 (13)
No	28.9 (13)	10.0 (3)
Distant metastasis			
M0	95.6 (43)	86.7 (18)	0.170
M1	2 (4.4)	13.3 (4)	
TNM stage			
Stage 1	75.6 (34)	53.3 (16)	0.061
Stage 2	2.2 (1)	6.7 (2)
Stage 3	8.9 (4)	3.3 (1)
Stage 4	13.3 (6)	36.7 (11)
ETA risk			
Low risk	42.2 (19)	27.6 (8)	0.168
Intermediate risk	11.1 (5)	6.7 (2)
High risk	46.7 (21)	66.7 (20)
RAI dose			
30–100 mCi	75.5 (34)	70 (21)	0.094
150 mCi	24.4 (11)	20 (6)
200 mCi	0	10 (3)
sTg			
Median (IQR)	3.05 (0.20–14.5)	140 (17.65–689)	<0.001
Preablation sTg			
≤18 ng	75.6 (34)	23.3 (7)	<0.001
>18 ng	24.4 (11)	76.6 (23)	
TG/TSH			
Median (IQR)	0.067 (0.005–0.26)	2.84 (0.366–22.61)	<0.001
sTg/TSH			
≤0.35	79.5 (35)	20 (6)	<0.001
>0.35	20.5 (9)	80 (24)	

**Table 2:** Cutoff values* for sTg and sTg/TSH ratio with sensitivity and specificity.

Predictor	Sensitivity	Specificity	Area under curve	*P* value
sTg				
≤18 ng/mL	76.7%	79.1%	0.86	<0.001
sTg/TSH ratio				
≤0.35	80%	81.4%	0.859	<0.001

*Values with maximum sensitivity and specificity.

**Table 3:** Univariate analysis of various patient and tumor characteristics and unsuccessful ablation.

Variable	OR (95% CI)	*P* value
Age		
40 or less	1	
More than 40	0.7 (0.28–1.77)	0.457
Gender		
Male	1	
Female	0.96 (0.38–2.43)	0.924
Size of tumor		
>4 cm	1	
<4 cm	2.02 (0.73–5.63)	0.176
Capsular invasion		
No	1	
Yes	2.71 (0.90–8.21)	0.077
Lymph node involvement		
No	1	
N1a	7.58 (1.31–43.92)	0.024
N1b	2.02 (0.43–9.6)	0.371
Nx	4.33 (0.99–18.87)	0.051
Distant metastasis		
M0	1	
M1	3.31 (0.57–9.34)	0.18
ETA risk		
Low risk	1	
Intermediate risk	0.95 (0.15–5.96)	0.956
High risk	2.26 (0.81–6.32)	0.120
sTg		
≤18 ng	1	
>18 ng	11.17 (3.71–33.61)	<0.001*
sTg/TSH		
≤0.35	1	
>0.35	15.56 (4.9–49.44)	<0.001*

*P-value < 0.005.

**Table 4:** Multivariate analysis of sTg levels, sTg/TSH ratio, and unsuccessful ablation.

Variable	AOR (95% CI)	*P* value
sTg		
≤18 ng	1	
>18 ng	5.42 (1.18–24.88)	0.030
sTg/TSH		
≤0.35	1	
>0.35	11.64 (2.43–55.61)	<0.001

AOR: adjusted odds ratio.

## Abbreviations

DTC: Differentiated thyroid carcinoma
RRA: Radioactive I^131^remnant ablation
TSH: Thyroid stimulating hormone
sTg: Stimulated thyroglobulin
AOR: Adjusted odds ratio
ATA: American Thyroid Association
Tg: Serum thyroglobulin
WBIS: Whole body I^131^ scan
PPV: Positive predictive value
NPV: Negative predictive value
Anti-Tg: Antithyroglobulin antibodies
ROC: Receiver operating curve
RAI: Radioactive I^131^
mCi: Millicurie
Gy: Gray
Rad: Radian
MBq: Megabecquerel
ETA: European Thyroid Association.
